# Functional Outcomes after Local Salvage Therapies for Radiation-Recurrent Prostate Cancer Patients: A Systematic Review

**DOI:** 10.3390/cancers13020244

**Published:** 2021-01-11

**Authors:** Mohammad Abufaraj, Abdelmuez Siyam, Mustafa Rami Ali, Rodrigo Suarez-Ibarrola, Lin Yang, Beat Foerster, Shahrokh F. Shariat

**Affiliations:** 1 Department of Urology, Medical University of Vienna, A-1080 Vienna, Austria; mabufaraj@ju.edu.jo; 2 Division of Urology, Department of Special Surgery, Jordan University Hospital, The University of Jordan, 11942 Amman, Jordan; ABD0150003@ju.edu.jo (A.S.); mustafa239@icloud.com (M.R.A.); 3 Department of Urology, Faculty of Medicine, University of Freiburg—Medical Centre, 79098 Freiburg, Germany; Rodrigo.suarez@uniklinik-freiburg.de; 4 Department of Cancer Epidemiology and Prevention, Cancer Care Alberta, Alberta Health Services, Calgary, AB T2S 3C3, Canada; lin.yang@ahs.ca; 5 Departments of Oncology and Community Health Sciences, Cumming school of medicine, University of Calgary, Calgary, AB T2N 4N1, Canada; 6 Department of Urology, Kantonsspital Winterthur, 8401 Winterthur, Switzerland; beat.foerster@bluewin.ch; 7 Institute for Urology and Human Reproductive Health, I.M. Sechenov First Moscow State Medical University, 119992 Moscow, Russia; 8 Department of Urology, University of Texas Southwestern Medical Center, Dallas, TX 75390, USA; 9 Department of Urology, Weill Cornell Medical College, New York-Presbyterian Hospital, New York, NY 10075, USA; 10 Department of Urology, Comprehensive Cancer Center, Medical University of Vienna, Vienna General Hospital, Währinger Gürtel 18-20, A-1090 Vienna, Austria

**Keywords:** salvage therapy, local therapy, radiation recurrence, prostate cancer, functional outcomes, morbidity

## Abstract

**Simple Summary:**

Local salvage therapies are offered to prostate cancer (PCa) patients with recurrent disease following primary radiation therapy with initial curative intent. Favorable oncologic outcome is the primary aim of salvage therapy, but many patients may be left with treatment-related adverse consequences, potentially affecting their quality of living. The aim of this review was to evaluate the rates and severity of various functional outcomes after salvage therapy in patients with radiation recurrent PCa. Local salvage therapies are associated with impaired urinary and sexual functions depending on the specific salvage strategy. However, accurate estimation of the likelihood of these sequalae may be predictable based on the high pre-salvage general and disease specific health status. Despite these adverse consequences and impaired quality of life, oncologic advantage of local salvage treatment post radiation recurrence prostate cancer seems justified in general, but shared decision working with an informed patient is essential. This paper serves as a discussion platform for this process.

**Abstract:**

Purpose: To assess the rate and severity of functional outcomes after salvage therapy for radiation recurrent prostate cancer. Methods: This systematic review of the MEDLINE/PubMed database yielded 35 studies, evaluating salvage radical prostatectomy (RP), brachytherapy (BT), high-intensity focal ultrasound (HIFU) and cryotherapy (CT) after failure of primary radiation therapy. Data on pre- and post-salvage rates and severity of functional outcomes (urinary incontinence, erectile dysfunction, and lower urinary tract symptoms) were collected from each study. Results: The rates of severe urinary incontinence ranged from 28–88%, 4.5–42%, 0–6.5%, 2.4–8% post salvage RP, HIFU, CT and BT, respectively. The rates of erectile dysfunction were relatively high reaching as much as 90%, 94.6%, 100%, 62% following RP, HIFU, CT and BT, respectively. Nonetheless, the high pre-salvage rates of ED preclude accurate estimation of the effect of salvage therapy. There was an increase in the median IPSS following salvage HIFU, BT and CT ranging from 2.5–3.4, 3.5–12, and 2, respectively. Extended follow-up showed a return-to-baseline IPSS in a salvage BT study. The reported data suffer from selection, reporting, publication and period of study biases, making inter-study comparisons inappropriate. Conclusions: local salvage therapies for radiation recurrent PCa affect continence, lower urinary tract symptoms and sexual functions. The use of local salvage therapies may be warranted in the setting of local disease control, but each individual decision must be made with the informed patient in a shared decision working process.

## 1. Introduction

The prevalence of prostate cancer (PCa) has been steadily increasing over the last few decades. The Global Burden of Disease study demonstrated a 3.7-fold rise in PCa incident cases between 1990 and 2015. This, together with the relatively modest PCa mortality and aging population, has generated a large pool of PCa survivors [1]. Despite the recent advances in PCa diagnosis and treatment, the burden of PCa remains significant, and PCa still constitutes the fifth most common cause of cancer mortality in the male population worldwide [2]. In addition, the economic burden of PCa management, in the United States alone, is considered the highest rising costs among all malignancies [3]. Furthermore, patients with PCa may suffer from disease- and treatment-related adverse events. [3].

There are several standard and widely used treatment options for patients with non-metastatic PCa, including external beam radiotherapy (EBRT), brachytherapy (BT), and radical prostatectomy (RP) [4]. Elderly patients with several comorbidities and limited life expectancy are less likely to undergo RP and consequently, with a proportionally increasing probability, receive radiation therapy (RT) [5,6]. Many other patients opt for RT because of other reasons, including the desire to avoid adverse events of RP [7,8]. The use of escalated dose radiotherapy is taking over conventional control-dose radiation as the results of randomized trials have shown that dose escalation has superior benefit in terms of biochemical recurrence (BCR) rates, local disease progression and distant metastasis [9,10]. Despite curative intent, a significant proportion of these patients ultimately experience PCa recurrence. Biochemical recurrence is estimated to affect 32–57% in control-dose RT and 16–43% in escalated dose RT after 10-years follow-up [10,11]. BCR is, in fact, a robust surrogate of clinical disease recurrence, including local recurrence and distant metastasis [12,13,14].

The rapid advancement in diagnostic imaging has improved the sensitivity of tumor staging and detecting small metastatic deposits, decreasing the number of patients diagnosed with isolated local recurrence [15]. Diagnosing local recurrence can be challenging due to radiation-induced changes in the prostate, compromising MRI findings, and histopathological evaluation [16,17].

Nonetheless, when local recurrence is detected, patients may benefit from local salvage therapies, including salvage RT, cryoablation, high-intensity focal ultrasound (HIFU), and RP [12]. However, salvage therapies are underutilized due to the limited high-quality data, unclear survival benefits, and treatment-related adverse effects [18,19,20]. These therapies might also be under-studied or simply not reported owing to the adverse consequences and lack of consensus on treatment algorithm. Androgen deprivation therapy (ADT) is, therefore, the most commonly utilized therapy in patients with radiation recurrent PCa [13], and the decision to pursue additional salvage interventions should balance the oncologic efficacy, adverse effects, the expertise as well as patient wishes and life expectancy [12].

Contemporary evidence on functional outcomes after local salvage therapies in patients with radiation recurrent PCa is unclear. We aimed to explore the functional outcomes and quality of life after local salvage therapies in patients with radiation recurrent PCa, and to create an evidence catalogue serving as a framework for research and shared decision making.

## 2. Materials and Methods

### Evidence Acquisition

This review followed the Preferred Reporting in Systematic Reviews and Meta-Analyses (PRISMA) protocol [21]. We conducted a systematic literature search using the PubMed-Medline database in July 2020, including articles published in the English language from January 2005 to June 2020. The search strategy included the following keywords in isolation or combination: “radio recurrent prostate cancer”, “radiation relapse in prostate cancer”, “radiation failure in prostate cancer”, “local salvage therapy”, “local therapy”, “salvage therapy [MeSH]”, “salvage cryoablation”, “salvage cryotherapy”, “salvage cryosurgery”, “salvage high-intensity focal ultrasound”, “salvage re-irradiation”, “salvage brachytherapy”, “salvage surgery”, “salvage prostatectomy” AND “functional outcomes”, “morbidity [MeSH]”, “adverse effects”, “patient-reported outcomes”, “clinical outcomes” and “quality of life [MeSH]”. The search was carried out by two authors independently, and any dispute was resolved by referring to a third author who acted as an arbiter.

The Population (P), Intervention (I), Comparator (C), Outcomes (O), and Study design (S) (PICOS) approach was used to specify the eligibility criteria. We considered a study eligible if it reported on PCa patients who were treated with primary radiation therapy (EBRT, BT, or both) and subsequently developed biochemical or clinical recurrence (P). Additionally, eligible studies should include data on patients treated by any local salvage therapy with or without ADT (I), and data on the treatment-related functional outcomes such as urinary function, sexual function, or quality of life (O) in prospective or retrospective studies (S). A comparator group (C) in each study was not necessary as an inclusion criterion since the outcomes of interest can be retrieved from case series or cohort studies and these outcomes can be compared across different studies.

We excluded review articles, case reports, articles reporting on ≤39 patients, comments, editorials, and conference abstracts. After duplicate removal, careful inspection of the remaining articles’ titles and abstracts was undertaken to rule out non-relevant articles. Studies involving multiple-modality primary therapies (except ADT) without separate analyses for radiation therapy as primary treatment were excluded. We also excluded studies reporting on oncological but not functional outcomes. An additional search in the references of all included studies was performed to screen for any articles that might have been missed in the primary search. All included articles were obtained as full-text articles for vigilant evaluation.

Reported urinary incontinence (UI) rates were classified as mild or severe. Mild UI was defined as any UI up to but not exceeding two pads per day, grade 1 (UI with coughing or sneezing) and 2 (UI with running or picking an object from the floor) using Ingelman-Sundberg UI [22], grade 1 (occasional UI, no pads needed) using CTCAE v4.0/v4.3 [23], and grade 1 (occasional, no pads needed) and 2 (pads needed, not interfering with ADL) using CTCAE v3.0 [24]. Severe UI was defined as any UI exceeding two pads per day, UI requiring instrumentation or surgery, grade 3 (UI with walking) using Ingelman-Sundberg UI [22], grade 2 (spontaneous UI, no intervention needed) and 3 (spontaneous, intervention needed) using CTCAE v4.0/v4.3 [23], and grade 3 (UI interfering with ADL) and 4 (surgery is needed) using CTCAE v3.0 [24].

Risk of bias assessment was performed using the National Institutes of Health (NIH) quality assessment tool for observational and cross-sectional studies [25]. This tool comprises 14 questions on each included study, addressing the quality of the study, the included cohort, ascertainment of exposure and outcomes of interest and follow-up data. A median follow-up period of 24 months post salvage therapy was defined as “adequate” for each included study. A point of “1” was given if the study fulfilled the information needed in the question. If not, a “0” point was given. If the question does not apply to the study or information not necessarily provided, a “N/A” score was given. As a total score, the summation of all the “1” scores was undertaken for each study.

## 3. Evidence Synthesis

### 3.1. Study Selection

**Figure 1:**
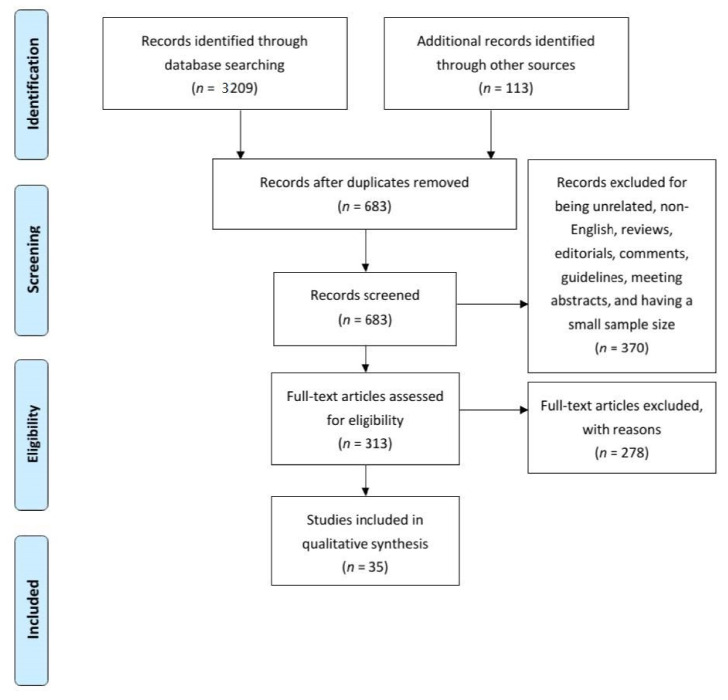
The Preferred Reporting in Systematic Reviews and meta-Analyses flowchart for article selection.

The primary search identified a total of 3209 articles while searching through additional sources yielded another 113 articles. After duplicates were removed, 683 articles remained, which were evaluated by reading the title and abstract of each. A subsequent full-text evaluation resulted in 35 articles to be included in evidence synthesis. Figure 1 shows the PRISMA flow chart and study selection process.

### 3.2. Study Characteristics

A total of 35 studies published between 2005 and 2020 met our inclusion criteria: 14 prospective [26,27,28,29,30,31,32,33,34,35,36,37,38,39] and 21 retrospective studies. Three studies evaluated surgery as salvage therapy [26,27,40], while one study [41] included surgery and HIFU as salvage modalities and provided separate analyses for each. A total of 246 patients underwent surgery as a salvage treatment modality. On the other hand, 31 studies evaluated other salvage interventions, with a total of 5018 patients. Primary treatment included a variation of EBRT alone, BT alone, a combination of both or proton beam therapy. EBRT was the sole primary modality in 13 studies [27,28,30,31,32,34,36,39,42,43,44,45,46], while BT was the only primary modality in one report [35]. In three studies, the primary treatment modality was not specified [37,47,48]. In the remaining 18 studies, more than one primary modality was used, and 15 studies of which reported the percentage of each modality. Of those 15, only nine studies reported that more than 70% of patients received primary EBRT [26,29,33,38,40,41,49,50,51,52,53,54,55,56,57,58,59,60]. Across all studies, ADT was initiated as adjuvant therapy in four studies [28,42,54,56], neoadjuvant therapy in seven studies [30,33,36,37,41,44,50], and both adjuvant and neoadjuvant in two studies [29,40]. Nineteen studies reported on ADT with no further details [26,31,32,34,35,39,43,45,46,48,49,51,52,53,55,57,58,59], and three studies did not report on the use of ADT [27,38,47]. Table 1 summarizes the characteristics of the included studies. The following functional outcomes after salvage intervention were extracted: UI, erectile dysfunction (ED), median/mean of the International Prostate Symptom Score (IPSS), and the International Index of Erectile Function (IIEF-5).

### 3.3. Urinary Incontinence

UI as a functional outcome of post-surgical salvage therapy was measured using the number of daily pads required following surgery. The reported UI rates at 12 months post-surgery ranged from 48% to 85% [26,27,40,41]. The rate of severe UI was more than 23% in all studies reporting on salvage surgery, with Mohler et al. and Seabra et al. reporting rates of 85% and 72%, respectively [26,27].

In studies reporting on non-surgical salvage interventions, continence was measured using scores such as the Ingelman-Sundberg score and the University of California Los Angeles (UCLA) urinary continence domain in addition to the number of pads. In the 13 studies that evaluated salvage HIFU, the reported UI rates ranged from 7.5% to 60.7% [31,32,33,34,35,36,41,42,44,45,46,56,57]. The rate of severe UI among these studies was less than 18% in eight studies, while Hostiou et al. and Jones et al. reported severe UI rates of 42% and 29%, respectively [31,35]. Three studies did not provide detailed information [32,44,57].

In patients who underwent cryotherapy after RT, the reported UI rates were between 3.2% and 52%, but two studies reported an UI rate of 0%: de Castro Abreu et al. in the focal cryotherapy group and Clarke et al. [28,37,38,47,49,50,51,52,58,59]. In addition, the reported rates of severe UI were less than 7%. Table 2 shows data on continence rates among included studies.

Patients who underwent salvage BThad UI rates ranging from 2.4% to 11% in four out of five studies (five out of six groups), while Henríquez López et al. reported an UI rate of 26.7% in the high dose rate BT group [29,30,43,54,55]. Three studies reported severe UI rates of 8%, 4.6%, and 2.4%, respectively [29,30,43].

### 3.4. Erectile Dysfunction

In patients who underwent salvage RP as their primary salvage modality, Mohler et al. and Seabra et al. reported ED rates of 78% and 74% at 6 months and 18 months, respectively [26,27]. In patients who underwent HIFU as the primary salvage modality, Hostiou et al., Jones et al. and Berge et al. reported ED rates of 76%, 53% and 94.6% during a median follow up of 12 months, 12 months, and 9 months, respectively [31,35,36]. Regarding studies assessing patients who underwent salvageBT, Van Son et al. demonstrated that 22% and 40% of their cohort experienced Grade II and III ED, respectively [29]. Studies on patients who underwent cryotherapy reported ED rates between 52% and 100% [28,37,39,47,48,50,51,59]. Furthermore, Robinson et al. and Donnelly et al. reported 3.5% and 4.3% unassisted intercourse rates in their cohort, respectively [28,39].

Studies that assessed non-surgical salvage modalities have also reported International Index of Erectile Function (IIEF) scores using the 5-item [29,33,44,57,58] and 15-item [32,35] questionnaires. Kanthabalan et al. demonstrated a decrease in the median IIEF from 15 to 13 in patients who received HIFU [57]. While other three studies report a decrease in mean (median) IIEF scores from 8.6 [6] to 6.2 [3], from 15.3 [9] to 8.3 [6] and from 13.2 [17] to 8.2 [20] in 43, 13 and 50 patients, respectively [32,35,44]. Siddiqui et al. reported a mean IIEF score of 8.6 preoperatively, and of 3.4, 5.1 and 5.4 at 1.5 months, 3 months, and 6 months, respectively, in their salvage HIFU group. Baco et al. reported a mean IIEF score of 11.2 preoperatively, and of 7.0 postoperatively [33,56].

Van Son et al. reported a median IIEF score of 11.0 preoperatively, and of 7.0 at 1 month and 3.0 at 36 months follow-up in patients who received BT [29]. Yamada et al. reported a decrease in the median IIEF score from 2.0 to 1.5 among 42 patients at 36 months follow-up [30].

Only one study reported IIEF scores in patients subjected to cryotherapy, in which Bomers et al. demonstrated a decrease in mean IIEF score from 11.7 to 9.0 in 44 patients [58]. Table 3 shows detailed information about ED in patients who underwent salvage interventions.

### 3.5. Urinary Obstruction

The International Prostate Symptoms Score (IPSS) was used as a tool to assess the lower urinary tract symptoms in patients who underwent several salvage interventions. In patients who underwent salvage HIFU, Hostiou et al. and Ahmed et al. reported an increase in mean (median) IPSS from 5.6 [4] to 8.1 (7.4) and from 8.3 [7] to 11.6 (9.5) in 50 and 46 patients, respectively [35,44]. Baco et al. reported an increase in mean IPSS from 7.1 to 8.6 in 47 patients [33].

In 50 patients who underwent BT, Van Son et al. reported a median IPSS of 8.0, 11.5 and 8.0 preoperatively, at 1-month postoperatively and 36 months postoperatively, respectively [29]. Kollmeier et al. reported a median preoperative IPSS of 7.0 and a median IPSS peak of 19.0 at 4 months follow-up [55]. Additionally, Yamada et al. reported a rise in median IPSS from 6.0 to 12.0 at 36 months in 42 patients [30].

Regarding salvage cryotherapy, Bomers et al. reported a mean preoperative IPSS of 9.0 and 10.2 at 12 months [58]. Lastly, Ismail et al. reported a median IPSS of 7.0 preoperatively, and 9.0 at 12 months follow-up.

### 3.6. Additional Outcomes

Other measured outcomes included the European Organization for Research and Treatment of Cancer Quality of Life Questionnaire Core Module (EORTC-QLC), the Research and Development short form 36 (RAND-SF 36), the International Continence Society questionnaire A and B (ICS (A), ICS (B)) and the University of California, Los Angeles Prostate Cancer Index (UCLA-PCI). Hostiou et al. reported an increase in mean (median) EORTC-QLC score from 33.6 [32] to 36.2 [34], while Baco et al. reported an increase in mean score from 35.7 to 36.8 [33,35]. Ahmed et al. reported a decrease in mean (median) RAND-SF 36 score from 102.7 (103) to 100.4 (100) at 6 months post-HIFU [44]. Additionally, Baco et al. reported an increase in mean ICS (A) and ICS (B) scores from 0.7 and 0.6 to 2.3 and 1.6, respectively [33]. Furthermore, Robinson et al. reported a decrease in mean UCLA-PCI scores of both urinary function and sexual function from 92 and 30 to 58 and 8 at 24 months post-cryotherapy, respectively, in 40 patients [39]. On the other hand, Berge et al. demonstrated deteriorating sexual function scores from 32.1 to 17.2 during follow-up of 17.5 months, as reported by the UCLA-PCI Short Form [46].

## 4. Risk of Bias Assessment

Other than Berge et al. [46], none of the included studies used a comparator group. Thus, the question on blinding the outcome assessors did not apply to the included studies and is thus given a “N/A” score (Question 12). The mean (median) bias scores of the studies were 8.6 [9], respectively. Most included studies showed an “intermediate” risk of bias, as 76% of the studies had a score 8–10 (Table 4).

## 5. Discussion

The treatment of radiation recurrent PCa represents a challenge given the lack of consensus on patients’ selection, heterogenous efficacy of local salvage modalities, and their variable toxicity profiles. It is often the last opportunity for local disease control and prevention of local and distant progression with its sequalae. The current review reports a relatively high prevalence of adverse functional outcomes following local salvage treatment for radiation recurrent PCa. It is worth noting that the pre-salvage rates of urinary and sexual dysfunction were generally high, probably owing to the effect of primary treatment. It is difficult to accurately determine the effect of salvage modalities on functional outcomes due to the significant heterogeneity among included studies, as well as the lack of standardized reporting methods and tools. Compared to primary RP, salvage RP is associated with a higher risk of complications such as ED, anastomotic stricture, urinary retention, urinary fistula, abscess, and rectal injury [61]. We observed high UI rates post salvage surgery, ranging from 48% to 85%, with severe UI rates exceeding those of mild UI. In addition, the rates of ED are high post salvage surgery. With longer follow-up, however, UI and ED rates declined. Moreover, the series were mostly older with significant changes in surgical technique and post-operative follow-up. Nonetheless, the relatively high preoperative rates preclude an accurate estimation of the effect of surgery, and limited follow-up of the studies hamper any long-term conclusions.

We found that ED rates are high, with at least half of men undergoing a local salvage treatment reporting ED. Interestingly, there was a high rate of ED before salvage therapy, with as much as 70% reporting ED. In a previous systematic review, Chade et al. found that 50–91% of patients had ED prior to salvage RP and 80–100% reported ED following salvage surgery [19]. In addition, urinary continence ranged between 21% and 90% after salvage surgery on longer follow up [19]. The variability of the findings may be mainly due to patient selection and surgical technique; in more recent series, these complications appear to be much less common, due to the progress in surgical technique and patient selection [62].

In patients who underwent salvage cryotherapy, we found that most of the included studies reported modest UI rates, mostly being mild. Two studies reported UI rates of 0% [38,51]. In a retrospective study of 143 patients who underwent cryotherapy after RT failure, Cespedes et al. reported high long-term UI rates of 28% at least 12 months after salvage cryotherapy, with 8–40% of patients reporting persistent rectal pain and 4% needing to undergo surgical procedures for the management of treatment-associated complications [63]. Notably, the introduction of cryotherapy has significantly decreased complications such as UI, fistulae, obstruction and ED [64]. For instance, in a recent study comprising only 14 patients, Boissier et al. reported de novo UI and ED in one patient, respectively [64]. Ideally, salvage cryotherapy has been suggested to be considered only for patients with comorbidities, a life expectancy of at least 10 years, an initial clinical stage of T1/T2, initial ISUP grade ≤ 2/3, a pre-salvage PSA-DT ≥ 16 months and a pre-salvage PSA ≤ 10 ng/mL [65].

High dose rate and low dose rateBT have been shown to be effective treatment options for histologically proven local PCa recurrence after RT with moderate Grade 3–4 gastrointestinal and genitourinary toxicity profiles ranging from 2.7–20% and 3–47%, respectively [66]. Overall, most salvage BT studies reported a 2.4% to 26.7% UI rate exceeding 1 pad daily. A meta-regression analysis comparing functional outcomes of surgical and non-surgical salvage modalities in radiation recurrent PCa found that both salvage BT and cryotherapy had significantly better results in terms of continence than salvage RP [67]. A subgroup analysis of patients who underwent non-surgical salvage modalities revealed that salvage BT and cryotherapy were comparable in UI rates. At the same time both were significantly better than salvage HIFU [67].

In terms of erectile function, the included studies illustrate the high rates of ED in this patient population, as shown by the decrease in IIEF scores compared to before therapy. However, a high-level of heterogeneity was noticeable between surgical and non-surgical salvage modalities indicating substantial differences between studies.

Salvage HIFU has recently emerged as an alternative thermal ablation option for radiation recurrent PCa [65]. In this review, most salvage HIFU studies reported continence rates above 50%. A meta-analysis showed that salvage HIFU did not demonstrate significantly better continence rates than salvage RP [67]. In general, the median follow-up did not exceed 24 months, a relatively adequate follow up period to report changes in outcomes [36,45]. Currently, there is a lack of high-quality data precluding robust recommendations regarding the indications for salvage HIFU.

We found considerable variability of functional outcomes after salvage therapy of radiation recurrent PCa. Proper patient selection and thorough consideration of the oncologic outcomes are critical factors in patient counselling and decision-making to achieve durable cancer control with the best possible quality of life.

In this review, we believe that the limitations of our work mainly stem from the heterogeneity of included studies, which even precluded a proper quantitative analysis. This review was based on case series and small cohort studies that lack a comparator group. The lack of extended follow-up duration was an issue only for some studies with outcomes as ED, whose rates might significantly change with time. Furthermore, this review focused on pertinent urologic functional outcomes, without analyzing other therapy-related adverse consequences such as GI toxicity. The significant heterogeneity of reporting outcomes and lack of pre-salvage rates of key outcomes also added to the problem. Therefore, it is difficult to draw solid conclusions with a high level of evidence. Indeed, prospective and comparative studies between different salvage modalities with long follow-up duration are needed to generate reliable evidence and validate long-term functional outcomes.

## 6. Conclusions

Local salvage therapies after radiation recurrent PCa are associated with impaired urinary and sexual functions. Accurate estimation of the impact of these therapies is precluded by the preoperative morbidity associated with primaryRT. Despite these adverse consequences, the oncologic advantage may justify the use of local salvage therapy post radiation failure in select informed patients who benefit from a balanced shared decision-making process.

## Figures and Tables

**Table 1:** Characteristics of studies assessing the functional outcomes in patients who underwent salvage intervention after radiation recurrent prostate cancer.

Name	Study Type	Location	Sample Size	Primary Treatment	Androgen Deprivation Therapy	Salvage Modality	Reported Outcomes
Surgical							
Mohler 2019 [26]	Prospective	USA	41	58.5%: EBRT 26.8%: BT 14.6%: Combined	22%: after recurrence	RP	Continence Potency
Devos 2019 (a) [41]	Retrospective	Belgium	25	68%: EBRT 32%: BT	36%: N-AD	RP	Continence
Seabra 2009 [27]	Prospective	Brazil	42	EBRT	-	RP	Continence Potency
Ward 2005 [40]	Retrospective	USA	138 (49: 1967–1990) (89: 1990–2001)	92%: EBRT 7.2%: BT 0.8%: Combined	38%: N-AD 61%: AD	RP	Continence
HIFU							
Hostiou 2019 [35]	Prospective maintenance, Retrospective analysis	France	50	BT	16%: at biochemical failure	HIFU	Continence Potency IPSS IIEF QOL (EORTC QLC-28)
Devos 2019 (b) [41]	Retrospective	Belgium	27	EBRT	31%: N-AD	HIFU	
Crouzet 2017 [45]	Retrospective	France	418	EBRT	none within 3 months of HIFU	HIFU	Continence
Jones 2018 [31]	Prospective trial	USA	100	EBRT	none within 3 months of HIFU	HIFU	Continence Potency
Kanthabalan 2017 [57]	Retrospective	UK	150	96.7%: EBRT 3.3%: EBRT + BT	45.3%: pre-salvage	HIFU	Continence IIEF
Shah 2016 [32]	Prospective	UK	50	EBRT	52%: after biochemical failure	HIFU	Continence IIEF
Siddiqui 2015 (a) [56]	Retrospective	UK	65	93.8%: EBRT 6.2%: BT	21%: AD	HIFU	Continence IIEF
Baco 2014 [33]	Prospective	France & Norway	48	95.8%: EBRT 4.2%: BT	22.9%: N-AD	HIFU	Continence IPSS IIEF ICS (A) ICS (B) EORTC QLC-30
Crouzet 2012 [34]	Prospective	France	290	EBRT	50%: prior to HIFU	HIFU	Continence
Berge 2011 [46]	Retrospective	Norway	61	EBRT	19%	HIFU	UCLA-PCI
Ahmed 2011 [44]	Retrospective	Canada and UK	84	EBRT	35.4%: N-AD	HIFU	Continence IPSS IIEF RAND-SF 36
Berge 2010 [36]	Prospective	Norway	46	EBRT	17.4%: N-AD	HIFU	Continence Potency
Murat 2009 [42]	Retrospective	France	167	EBRT	56.8%: post primary therapy or AD	HIFU	Continence
Cryotherapy							
Bomers 2020 [58]	Retrospective	Netherlands	62	64.5%: EBRT 33.9%: BT 1.6%: Combined	prior use: 37.1%	MR imaging–guided CT	IPSS IIEF
Tan 2020 (a) [59]	Retrospective	COLD registry	385	75%: EBRT 16%: BT 9%: Combined	26.4%	Focal CT: 72 patients	Continence Potency
Tan 2020 (b) [59]	Retrospective	COLD registry	385	75%: EBRT 16%: BT 9%: Combined	31.3%: prior to salvage	Total CT: 313 patients	
Safavy 2019 [60]	Retrospective	USA	75	77.3%: EBRT 21.3%: BT 1.3%: missing	25.3%: were subjected to ADT	CT	Continence
Siddiqui 2016 [49]	Retrospective	Canada	157	EBRT or EBRT + BT or BT	71%: prior to salvage	CT	Continence
Li 2015 [48]	Retrospective	COLD registry	740	Radiotherapy	34.3%: prior to salvage	CT	Continence Potency
Siddiqui 2015 (b) [56]	Retrospective	UK	65	97%: EBRT3%: BT	13%: AD	CT 1995–1998	
Siddiqui 2015 (c) [56]	Retrospective	UK	65	EBRT	18%: AD	CT 2002–2004	
Li 2014 [50]	Retrospective	COLD registry	91	25: BT 44: EBRT 3: Combined	35.2%: N-AD	CT	Continence Potency
Ahmad 2013 [47]	Retrospective	UK	283	Radiotherapy	-	CT	Continence Potency
De Castro Abreu 2013 (a) [51]	Retrospective	USA	25	44%: EBRT 32%: Proton beam 20%: BT 4%: BT + EBRT	none during study period before recurrence	Focal CT, UL	Continence Potency
De Castro Abreu 2013 (b) [51]	Retrospective	USA	25	44%: EBRT 20%: Proton beam 28%: BT 8%: BT + EBRT	none during study period before recurrence	Total CT, BL	
Pisters 2008 [52]	Retrospective	USA	279	78.1%: EBRT 11.5%: BT 7.2%: Combined 3.2%: unknown	50.9%: prior to salvage	CT	Continence
Ismail 2007 [37]	Prospective case series	UK	100	Radiotherapy	46%: N-AD	CT	Continence PotencyIPSS
Ng 2007 [53]	Retrospective	USA	187	97.9%: EBRT 1.6%: BT 0.5%: Combined	32%: started ADT due to disease progression	CT	Continence
Clarke 2007 [38]	Prospective	USA	47	EBRT or BT or both	-	CT	Continence
Robinson 2006 [39]	Prospective PHASE II study	Canada	46	EBRT	26.1%: pre cryosurgery 15.2% post crysurgery	CT	Potency UCLA-PCI
Donnelly 2005 [28]	Prospective	Canada	46	EBRT	6.5%: AD	Ultrasound-guided CT	Continence Potency
Brachytherapy							
van Son 2020 [29]	Prospective	Netherlands	50	50%: EBRT 50%: BT	8%: N-AD 14%: AD	BT	Continence Potency IPSS IIEF
Crook 2019 [43]	Retrospective	Canada	92	EBRT	16%: at study entry	BT	Continence
Lopez 2019 (a) [54]	Retrospective	Spain	73	EBRT or BT	29%: AD	HDR BT	Continence
Lopez 2019 (b) [54]	Retrospective	Spain	44	EBRT or BT	18%: AD	LDR BT	
Kollmeier 2017 [55]	Retrospective	USA	98	87.8%: EBRT 10.2%: BT 2%: Combined	45%: at salvage	37.8%: LDR BT 62.2%: HDR BT	Continence IPSS
Yamada 2014 [30]	Prospective PHASE II study	USA	42	EBRT	43%: N-AD	BT	Continence IPSS IIEF

P = prospective, R = retrospective, [*n*] = number of patients assessed in given outcome, EBRT = external beam radiation therapy, BT = brachytherapy, RP = radical prostatectomy, AD = adjuvant, N-AD = neoadjuvant, HRQOL = health related quality of life, HIFU = high intensity focused ultrasound, CT = cryotherapy, ADT = androgen deprivation therapy, COLD = Cryo On-Line Data Registry, HDR = high dose rate, LDR = low dose rate, 3D-CT = three dimensional computed tomography, IPSS = international prostate symptom score, IIEF = international index of erectile function, EORTC QLC = European Organization for Research and Treatment of Cancer Quality of Life Questionnaire Core Module, ICS (A) = International Continence Society questionnaire A, ICS (B) = International Continence Society questionnaire B, UCLA-PCI = University of California, Los Angeles Prostate Cancer Index, RAND-SF 36 = RAND short form 36.

**Table 2:** Urinary function outcomes after various salvage interventions presented as mild versus severe urinary incontinence.

Author and Year of Publication	Functional Outcomes Sample	Incontinence Rate (General)	Mild UI ^¥^	Severe UI ^£^
Surgery				
Mohler 2019 [26]	40 (6 months) 34 (12 months) 32 (24 months) 24 (36 months)	-	-	88% 85% 63% 42%
Devos 2019 (a)	25	56%	28%	28%
Seabra 2009 [27]	42	72%	-	72%
Ward 2005 [40]	130	48%	At least 20% **	Less than 28% **
HIFU				
Hostiou 2019 [35]	50	-	14%	42%
Devos 2019 (b)	27	22%	11%	11%
Crouzet 2017 [45]	388	49%	33%	16%
Jones 2018 [31]	100	47%	18%	29%
Kanthabalan 2017 [57]	48	22% *	-	-
Shah 2016 [32]	26	31% *	-	-
Siddiqui 2015 (a) [56]	65	7.50%	3%	4.5%
Baco 2014 [33]	48	25.30%	17%	8.3%
Crouzet 2012 [34]	290	46%	37%	16.8%
Berge 2011 [46]	61	-	44%	16%
Ahmed 2012 [44]	84	38%	N/A ^§^	N/A
Berge 2010 [36]	35	60.7%	43.4%	17.3%
Murat 2009 [42]	167	49.50%	40%	9.5%
Cryotherapy				
Bomers 2020 [58]	44	3.2%	-	3.2%
Tan 2020 (a) ^₡^ [59]	72	9.30%	N/A	N/A
Tan 2020 (b) ^₡^ [59]	313	15.10%	N/A	N/A
Safavy 2019 [60]	75	25.30% *	-	-
Siddiqui 2016 [49]	157	48%	44%	4%
Li 2015 [48]	740	No pre-SC ADT: 33.3% * With pre-SC ADT: 23.3% *	-	-
Siddiqui 2015 (b) [56]	65	52%	49%	3%
Siddiqui 2015 (c) [56]	65	37.50%	31%	6.5%
Li 2014 [50]	91	5.50% *	-	-
Ahmad 2013 [47]	283	12% *	-	-
De Castro Abreu 2013 (a) [51]	25	0% *	-	-
De Castro Abreu 2013 (b) [51]	25	13% *	-	-
Pisters 2008 [52]	137	10.20% *	-	-
Ismail 2007 [37]	100	13%	Less than 7% **	At least 6% **
Ng 2007 [53]	187	40% +	37%	3%
Clarke 2007 [38]	47	0%	0%	0%
Donnelly 2005 [28]	46	6.50%	N/A	N/A
Brachytherapy				
Van Son 2020 [29]	50	8%	-	8%
Crook 2019 [43]	87	4.6%	-	4.6%
Henríquez López 2019 (a) [54]	73	26.70% *	-	-
Henríquez López 2019 (b) [54]	44	4.50% *	-	-
Kollmeier 2017 [55]	98	11% *	-	-
Yamada 2014 [30]	42	2.40%	-	2.4%

^¥^: mild UI was defined as any UI up to but not exceeding two pads per day, grade 1 (UI with coughing or sneezing) and 2 (UI with running or picking an object from the floor) Ingleman-Sundberg UI, grade 1 (occasional UI, no pads needed) CTCAE v4.0/v4.3, and grade 1 (occasional, no pads needed) and 2 (pads needed, not interfering with ADL) CTCAE v3.0. £: severe UI was defined as any UI exceeding two pads per day, UI requiring instrumentation or surgery, grade 3 (UI with walking) Ingleman-Sundberg UI, grade 2 (spontaneous UI, no intervention needed) and 3 (spontaneous, intervention needed) CTCAE v4.0/v4.3, and grade 3 (UI interfering with ADL) and 4 (surgery is needed) CTCAE v3.0 ^§^: no further details on pads or grades provided. ^₡^: Tan (a) used focal CT, while Tan (b) used total CT *: ≥1 pad with no other specifications **: the numbers reported do not match those of our table (e.g., <3 pads and ≥3 pads); the lower limit underestimates UI and the upper limit overestimates UI, so “less than” and “at least” were added accordingly. +: “Mild to moderate” was put as (mild UI), “severe” was put as (severe UI), [*n*] = number of patients assessed for given outcome, UCLA-PCI = University of California, Los Angeles Prostate Cancer Index.

**Table 3:** Sexual function outcomes in patients who underwent various salvage interventions.

Author and Year of Publication	Functional Outcomes Follow-Up Sample	Pre-op ED Rate	Post-op ED Rate	Follow-Up Time for EF (Months)
Surgical				
Mohler 2019 [26]	40 at 3 months 40 at 6 months 24 at 36 months	32% [38]	90% at 3 months 78% at 6 months 25% at 36 months	-
Seabra 2009 [27]	42	-	74%	-
HIFU				
Hostiou 2019 ^A^ [35]	50	50%	76%	12
Jones 2018 [31]	100	53%	88%	12
Kanthabalan 2017 ^B^ [57]	48	-	-	-
Shah 2016 ^A^ [32]	26	-	-	-
Siddiqui 2015 (a) [56]	65	-	-	-
Baco 2014 ^B^ [33]	48	-	-	-
Ahmed 2012 ^B^ [44]	84	-	-	-
Berge 2010 [36]	37	78.8% [33]	94.6%	-
Cryotherapy				
Bomers 2020 ^B^ [58]	44	-	-	-
Tan 2020 (a) ^¥^ [59]	72	-	52.60%	12
Tan 2020 (b) ^¥^ [59]	313	-	59.60%	12
Li 2015 [48]	740	-	No pre-SC ADT: 71.3% With pre-SC ADT: 84.5%	12
Li 2014 [50]	91	70%	85%	-
Ahmad 2013 [47]	283	-	83%	-
De Castro Abreu 2013 (a) [51]	25	72%	92%	-
De Castro Abreu 2013 (b) [51]	25	84%	100%	-
Ismail 2007 [37]	63	-	86%	-
Robinson 2006 [39]	40	67.90%	86.20%	24
Donnelly 2005 [28]	46	72%	84.90%	6
Brachytherapy				
Van Son 2020 ^B^ [29]	50	18% grade III	40% grade III 22% grade II	-
Yamada 2014 [30]	42	-	-	-

[*n*] = number of patients assessed for given outcome, ^A^ = IIEF-15 was used, ^B^ = IIEF-5 was used, ^¥^: Tan (a) used focal CT, while Tan (b) used total CT. IIEF-5 = international index of erectile function 5-point scale, IIEF-15 = international index of erectile function 15-point scale, UCLA-PCI = University of California, Los Angeles Prostate Cancer Index.

**Table 4:** Risk of Bias assessment of included studies using the Quality Assessment Tool for Observational Cohort and Cross-Sectional Studies, NIH.

Study	Q1	Q2	Q3	Q4	Q5	Q6	Q7 *	Q8	Q9	Q10	Q11	Q12	Q13	Q14	Total
Ahmad 2013 [47]	1	1	1	1	0	1	1	1	1	0	1	N/A	1	1	11
Ahmed 2011 [44]	1	1	1	1	0	1	0	0	1	0	1	N/A	0	1	8
Baco 2014 [33]	1	1	1	1	0	1	0	0	1	0	1	N/A	1	0	9
Berge 2010 [36]	1	1	1	1	0	1	0	0	1	0	1	N/A	1	0	9
Berge 2011 [46]	1	1	1	1	0	1	0	0	1	0	1	0	1	1	10
Bomers 2020 [58]	1	1	1	1	0	1	0	0	1	0	1	N/A	1	1	10
Clarke 2007 [38]	1	1	1	1	0	1	1	0	1	0	0	N/A	1	1	9
Crook 2019 [43]	1	1	0	1	1	1	1	0	1	0	1	N/A	1	1	10
Crouzet 2012 [34]	1	1	0	1	0	1	1	0	1	0	1	N/A	1	1	9
Crouzet 2017 [45]	1	1	0	1	0	1	1	0	1	0	1	N/A	1	1	9
De castro Abreu 2013 [51]	1	1	0	1	0	1	1	1	1	0	0	N/A	1	0	8
Devos 2019 (HIFU) [41]	1	1	0	1	0	1	1	0	1	0	1	N/A	1	0	8
Devos 2019 (RP) [41]	1	1	0	1	0	1	1	0	N/A	0	1	N/A	1	0	7
Donnelly 2005 [28]	1	1	0	1	0	1	0	0	1	0	0	N/A	1	0	7
Hostiou 2019 [35]	1	1	1	1	0	1	1	1	1	0	1	N/A	1	1	11
Ismail 2007 [37]	1	1	1	1	0	1	0	0	1	0	1	N/A	1	1	10
Jones 2018 [31]	1	1	0	1	0	1	0	0	1	0	1	N/A	1	0	8
Kanthabalan 2017 [57]	1	1	1	1	1	1	1	0	1	0	1	N/A	0	1	10
Kollmeier 2017 [55]	1	1	0	1	0	1	0	0	1	0	1	N/A	1	1	8
Li 2014 [50]	1	1	0	1	0	1	0	0	1	0	0	N/A	1	1	8
Li 2015 [48]	1	1	1	1	1	1	0	0	1	0	0	N/A	1	1	10
Lopez 2019 [54]	1	1	0	1	1	1	1	1	1	0	1	N/A	1	1	11
Mohler 2019 [26]	1	1	1	1	0	1	1	N/A	N/A	0	0	N/A	1	0	7
Murat 2009 [42]	1	1	0	1	0	1	0	0	1	0	1	N/A	1	1	9
NG 2007 [53]	1	1	0	1	0	1	1	0	1	0	0	N/A	1	1	8
Pisters 2008 [52]	1	1	1	1	0	1	0	0	1	0	1	N/A	0	0	8
Robinson 2006 [39]	1	1	1	1	0	1	1	0	1	0	1	N/A	1	0	9
Safavy 2019 [60]	1	1	1	1	1	1	1	0	1	0	1	N/A	1	0	10
Seabra 2009 [27]	1	1	0	1	0	1	0	N/A	N/A	0	0	N/A	1	0	6
Shah 2016 [32]	1	1	1	1	1	1	0	0	1	0	1	N/A	0	1	10
Siddiqui 2015 [56]	1	1	0	0	0	1	0	1	1	0	1	N/A	1	0	7
Siddiqui 2016 [49]	1	1	0	1	0	1	1	0	1	0	1	N/A	1	1	9
Tan 2020 [59]	1	1	0	1	1	1	0	1	1	0	0	N/A	1	1	10
Van Son 2020 [29]	1	1	1	1	0	1	1	0	1	0	1	N/A	1	1	10
Ward 2005 [40]	1	1	0	1	0	1	0	N/A	N/A	0	1	N/A	1	1	8
Yamada 2014 [30]	1	1	1	1	0	1	1	0	1	0	1	N/A	1	0	9

*: a median postoperative follow-up period of 24 months was considered adequate.

## Data Availability

Not applicable.
